# Fat Checking: Emerging Role of Lipids in Metabolism and Disease

**DOI:** 10.3390/ijms232213842

**Published:** 2022-11-10

**Authors:** Marco Segatto, Antimo Cutone, Valentina Pallottini

**Affiliations:** 1 Department of Biosciences and Territory, University of Molise, 86090 Pesche, Italy; 2 Department of Science, University Roma Tre, 00146 Rome, Italy

Lipids are hydrophobic molecules involved in a plethora of biological functions; for example, they are employed for the storage of energy, serve as essential constituents of cell membranes and participate in the assembly of bilayer configuration. Specifically, within membranes, lipids mainly accumulate in highly specialized sphingolipid- and cholesterol-rich domains, such as caveolae and lipid rafts, where they function as platforms for the recruitment of proteins involved in signaling cascades and cell–cell interactions [1]. Besides these structural roles, lipids can also regulate several cellular processes thorough acting as primary and secondary messengers. The interest in the metabolism of lipids is of particular relevance in biomedical research and clinical practice, because of their incontrovertible implications in a number of human disorders. For instance, hyperlipidemia is an important risk factor for the development of cardiovascular diseases [2], and other reports having demonstrated that cancer cells undergo metabolic changes in lipid homeostasis, a reprogramming that is essential to the sustainment of pivotal oncogenic functions [3,4]. Moreover, increasing evidence suggests that lipids play key roles in the central nervous system [5]. Thus, it is obvious why deregulations in the homeostasis of lipids are often associated with the onset of neurodegenerative conditions [6].

In recent years, research has allowed for significant advancements in knowledge surrounding the role of lipids in human health. However, many details remain unknown, necessitating more in-depth knowledge to help better understand pathogenetic mechanisms, as well as to identify novel therapeutic approaches.

This Special Issue, entitled “Emerging Role of Lipids in Metabolism and Disease–2nd Edition”, comprises twenty articles selected for publication.

As mentioned above, the maintenance of lipid homeostasis is essential to guarantee several brain processes. In their review article, Colardo et al. summarized the currently available knowledge linking neurotrophins and the lipid metabolism. Indeed, increasing evidence sustains that neurotrophins not only regulate classical signaling pathways involved in cell survival, growth and differentiation, but also control cell metabolism through influencing lipid homeostasis [7].

Oxylipins are bioactive lipid mediators derived from the oxidation of polyunsaturated fatty acids; they act as potent signaling molecules that regulate several biological processes, including neuroinflammation. Jonnalagadda et al. reported that 1-trifluoromethoxyphenyl-3-(1-propionylpiperidin-4-yl) urea (TPPU), a selective soluble epoxide hydrolase inhibitor, has shown the capacity to efficiently counteract pathological features of experimental autoimmune encephalomyelitis (EAE), a preclinical model of multiple sclerosis. Notably, the effects were mediated through a decrease in the production of the proinflammatory dihydroxy fatty acids, as well as a concurrent build up in anti-inflammatory epoxy fatty acids [8]. Other research groups focused their attention on innovative strategies for combatting neuroinflammation, regulating oxylipin production through the use of astrocytes. Chistyakov et al. showed that peroxisome proliferator-activated receptor (PPAR) ligands, particularly PPARβ agonists, strongly reduced COX-derived oxylipins and suppressed the release of proinflammatory cytokines in lipopolysaccharide (LPS)-stimulated primary rat astrocytes [9]. Interestingly, the same research group also highlighted that another approach to reducing the production of oxylipins and proinflammatory cytokines due to LPS-stimulated astrocytes was the administration of 4-methylumbelliferone, a specific inhibitor of hyaluronic acid synthesis [10].

Conte et al. shed light on the role played by lipids in cell-to-cell communication, having found exosomes released from HN9.10 embryonic hippocampal cells to be rich in sphingomyelin species. Notably, this effect was associated with an increase in the neutral sphingomyelinase content induced through the administration of vitamin D3. Ceramide-enriched exosomes are necessary in the promotion of neuronal differentiation, suggesting vitamin D3 drives cell differentiation through the modulation of the amount of ceramide within the exosomes [11]. Alterations in sphingolipid profile could be associated with several neuropathological conditions; for instance, Torretta et al. showed that very-long-chain ceramides increase in idiopathic normal pressure hydrocephalus, suggesting that these lipids could potentially be considered as promising biomarkers for this disease [12].

Lipids have recently attracted increasing interest in cancer biology due to their potential role in mechanisms responsible for controlling cell growth, proliferation and survival. Hofmanová et al. highlighted that isolated colon cancer epithelial cells are characterized by an increased expression of fatty acids, an event that can be explained by the sustained expression of genes involved in fatty acid biosynthesis, elongation and desaturation. The authors concluded that the enhanced de novo synthesis of very-long-chain fatty acids, as well as the deregulations of their incorporation into specific lysophospholipids, may take part in the reprogramming of phospholipidome, which, in turn, may determine tumor heterogeneity and response to therapies [13]. Lipid reprogramming is not a prerogative of colon cancer, since a number of other tumors, such as hepatocellular carcinoma, are characterized by a shift in the lipid metabolism. In order to better comprehend the impact of lipids in cancer physiopathology, Haberl et al. focused their attention on lipid alterations in nonmalignant and tumor hepatic tissues as a function of hepatitis B virus (HBV) and hepatitis C virus (HCV) infections, suggesting that the disease etiology ought to be considered when designing novel therapeutic approaches against the lipid metabolism in chronic viral hepatitis and associated cancers [14]. The involvement of lipids in cancer biology is further sustained with the study by Guth et al., demonstrating that the pharmacological or genetic blockade of lipid oxidation ameliorates the intercommunication between cancer and immune cells, favoring T-cell cytotoxic activity and, in turn, the antitumoral response [15]. Furthermore, the review article by Rysz et al. provides an extensive analysis of the connection between metabolic aspects, such as dyslipidemia, and the development and progression of renal cancers [16].

Two interesting review articles summarize the current understanding concerning the role of lipids in the immune system. Rosa Neto et al. focused their attention on the metabolic role fatty acids exert during the determination of the phenotype, activation and function of macrophages and lymphocytes [17]. On the other hand, Wójcik et al. described the pivotal role of lipid mediators, such as endocannabinoids and eicosanoids, in the modulation of inflammation in autoimmune diseases [18].

The proper functionality of skeletal muscle is guaranteed by the maintenance of lipid homeostasis. For instance, Bryndina et al. demonstrated that motor and afferent inputs govern ceramide distribution, thus, influencing the stability of lipid rafts in the junctional and extrajunctional membranes of muscle fibers [19]. Branched fatty acid esters of hydroxy fatty acids (FAHFAs) play regulatory roles in muscle physiology; Benlebna et al. provided evidence that these lipids inhibit myoblast proliferation and facilitate the fiber shift toward a slower phenotype by increasing the expression of myosin heavy-chain (MyHC) IIa and IIx isoforms [20].

The liver is considered the metabolic power station of the whole body; thus, it is not surprising that important processes controlling the lipid metabolism reside within this organ. Popeijus et al. revised the scientific literature to gather available information concerning the influence of short-chain fatty acids on the ApoA-I metabolism in hepatic cells [21]. Hepatic lipid metabolism deregulations are frequently associated with the development of nonalcoholic fatty liver disease (NFALD), a major health problem often leading to cirrhosis and hepatocellular carcinoma. The review by Hliwa et al. summarized the state-of-the-art regarding the involvement of lipid alterations in NAFLD, placing particular emphasis on disbalances in fatty acids [22].

Behl et al. focused their attention on the interesting relationship found between rheumatoid arthritis (RA) and cardiovascular disorders (CVDs). Since CVDs are often associated with lipid disorders, several research articles explored the possibility that the lipid metabolism can be affected in RA. In this review, the authors also collected current knowledge regarding this intricate association [23].

The review article provided by Barale et al. comprehensively summarizes the current understanding of the role exerted by proprotein convertase subtilisin/kexin type 9 (PCSK9) in the regulation of the lipid metabolism, focusing particular attention on the prospective therapeutic interventions based on PCSK9 inhibition [24].

The work by Stasi et al. describes the multiple roles that HDL may play in sepsis and SARS-CoV-2 infection, with particular references to renal implications [25].

Lastly, the review by Jin et al. explains the molecular basis and factors accountable for regulating the motility of lipid droplets [26].
